# Application of Construction and Demolition Waste in Civil Construction in the Brazilian Amazon—Case Study of the City of Rio Branco

**DOI:** 10.3390/ma14092247

**Published:** 2021-04-27

**Authors:** Fernando da Silva Souza, José Maria Franco de Carvalho, Gabriela Grotti Silveira, Vitória Cordeiro Araújo, Ricardo André Fiorotti Peixoto

**Affiliations:** 1 Laboratory of Materials for Civil Construction, Department of Civil Engineering, Federal University of Ouro Preto, 35400-000 Ouro Preto, Brazil; ricardofiorotti@ufop.edu.br; 2 Laboratory of Materials for Civil Construction, Department of Civil Engineering, Federal University of Acre, 69920-900 Rio Branco, Brazil; gabigrotti.gg@gmail.com (G.G.S.); engvitoriacordeiro@gmail.com (V.C.A.); 3 Laboratory of Composite Materials, Department of Civil Engineering, Federal University of Viçosa, 36570-900 Viçosa, Brazil; josemaria.carvalho@ufv.br

**Keywords:** construction and demolition waste, recycled aggregate, sustainable development, Amazon region, eco-efficient cement composite

## Abstract

The lack of usable aggregates for civil construction in Rio Branco (capital of Acre, a Federal State in the Amazon region) makes the production and use of recycled aggregates from construction and demolition waste (CDW) an alternative of great interest. In this study, a comprehensive characterization of CDW collected from 24 construction sites of six building types and three different construction phases (structures, masonry, and finishing) was carried out. The fine and coarse recycled aggregates were produced and evaluated in 10 different compositions. The aggregates’ performance was evaluated in four mixtures designed for laying and coating mortars with a total replacement of conventional aggregates and a mixture designed for a C25 concrete with 50% and 100% replacement of conventional aggregates. CDW mortars showed lower densities and greater water retention, initial adhesion, and mechanical strength than conventional mortars. CDW concretes presented lower densities and greater resistance to chloride penetration than conventional concrete, with a small mechanical strength reduction. The recycled CDW aggregates proved to be technologically feasible for safe application in mortars and concrete; for this reason, it is believed that the alternative and proposed methodology is of great interest to the Amazonian construction industry, considering the high costs of raw materials and the need for defining and consolidating a sustainable development model for the Amazon region.

## 1. Introduction

The civil construction industry represented 3.8% of Brazil’s Gross Domestic Product (GDP) in 2019, with 21.4% of the Brazilian industry’s total market that fosters the country’s development and technologies [1]. However, the non-reuse of the generated construction and demolition waste (CDW) causes environmental, economic, and social impacts, representing a serious problem in several cities worldwide.

CDW represents more than one-third of the waste generated in the European Union (EU), with a variation in recycling levels between countries from less than 10% to more than 90% [2]. In 2018, 368 million tons of CDW (CDW mineral waste—except soils) were generated, with Germany (86 million), the United Kingdom (69 million), and France (69 million) the largest generators [3]. The United States was responsible for the generation of 534 million tons of CDW (in 2014), while China has the largest generation of CDW in the world of 1.13 billion tons (in 2014) [4].

It is estimated that 35% of the global CDW is buried, and effective management is necessary to minimize environmental impacts [5]. Research shows that recycling the CDW brings environmental benefits, with the collection and sorting at the origin of the generation being fundamental actions to reduce carbon emissions [6,7]. CDW recycling leads to significant reductions in emissions, energy use, global warming potential (GWP), and conserves landfill spaces [8].

The Brazilian law No. 12305 (National Solid Waste Policy) [9] and CONAMA Resolution 307/2002 [10] establish guidelines, criteria, and procedures for the management of CDWs, where the municipal public authority becomes responsible for establishing actions for the correct management and directing the construction companies to properly dispose of the waste generated, aiming to mitigate the impacts caused by their irregular disposal.

Civil construction in the city of Rio Branco, located in the Brazilian Amazon, has the third-highest cost per square meter of construction in Brazil (in November 2020) [11]. This fact is justified by the geographic location in the far west of the country, raising the cost of construction materials due to transport logistics. Thus, Rio Branco has the most expensive construction material in the country, with prices 17.7% higher than the national average, 16.8% higher than those observed in the State of São Paulo, 17.4% higher than those observed in the State of Rio de Janeiro, 21% higher than those observed in the State of Minas Gerais, and 28% higher than those observed in the State of Espírito Santo (in November 2020) [11]. For example, cement was sold in October 2020 at US$9.20 (50 kg bag), which is 92% more expensive than São Paulo and Espírito Santo (US $4.8), 84% more than Rio de Janeiro (US$5.0), and 77% more than Minas Gerais (US$5.2) [12].

There is a lack of natural and quality aggregates for construction in the city of Rio Branco because the sand extracted from the bed of the Rio Acre presents a granulometric curve out of the lower usable limit prescribed by the Brazilian specification NBR 7211 [13], classifying it as very fine sand [14], requiring more water and cement in the usual mortar and concrete lines.

About the coarse aggregate, due to the geological formation, the state has no natural reserves, having to import from the nearest quarry that is 300 km distant from Rio Branco, located in the State of Rondônia, in the region of the Abunã River [15], greatly increasing the cost of input due to logistics by land and crossing the Madeira River by ferry. That makes the aggregates markedly expensive in the acre state (US$49.0/m³), which is, on average, 1.9 times more than in Rondônia (US$25.5/m³) and 3.4 times more than the southeastern state of Minas Gerais (US$14.2/m³), which is famous for its large number of quarries [12].

The reuse of construction waste as recycled aggregates reduces the volume destined to the landfill and the consumption of natural resources, providing less environmental degradation, which means less energy consumption for extraction, processing, and transportation. Several authors have shown the use of recycled aggregates in mortars and concrete with similar or even better properties than conventional materials [16,17,18,19,20,21,22,23,24,25,26].

Zordan [18], Leite [25], Chen, Yen, and Chen [24] concluded that recycled aggregates are technically feasible to be used in concrete. Gómez-Soberon [25] stated that even with the high porosity of recycled aggregates, it is possible to use them in concrete. Viera and Dal Molin [23] showed that recycled aggregates could improve some concrete properties, such as compressive strength and durability, which are measured by estimating its service life. Poon and Chan [27] showed that concrete with up to 20% substitution of conventional aggregate for recycled aggregate or 100% substitution with a slight increase in the cement content (4%) presented performances similar to conventional concrete. Jimenez et al. [19] tested five mortars with different replacement rates of natural sand by recycled aggregate (0%, 5%, 10%, 20%, and 40%), and the results showed that rates of up to 40% did not significantly affect the properties of fresh and hardened mortar. Ho et al. [21] showed that up to 100% substitution, without compensating for water absorption and using water-reducing admixtures, produced concretes with mechanical performance and durability equivalent to conventional concrete. Samadi et al. [16] showed that the use of ceramic waste as a binder and fine aggregate source significantly improved the mortar’s compressive strength and provided greater resistance to adverse environmental conditions. Recently, RC-Beton [20] reported that 2650 m³ of concrete containing up to 20% recycled aggregate, with strength classes C25/30 and C30/37, were produced and used to construct an office building in Germany. The concrete was applied in various structural elements of foundations, slabs, and walls, achieving the specified strength according to the project.

The literature shows that the use of recycled CDW aggregates is becoming a feasible and consolidated practice. However, a methodology for obtaining, characterizing, and applying recycled CDW aggregates based on the construction phase has not been found in the literature. This work is also the first in covering such methodological approach and application of recycled CDW aggregates in the Amazon region, which enhances its relevance by addressing very specific environmental and economic issues in a sensitive and very significant region to the world that is in its search for its efficient model of sustainable development. Additionally, the study area lacks data on the application of CDW in civil construction at a regional and national level. For this reason, the applied methodology can be used as a starting point for future studies in this area, and the data collected can be used by public or private policies to support tactical and strategic decisions for effective management in the recycling of CDW aimed at reuse in civil construction. It is worth mentioning that such studies are important to put pressure on the modernization of the current practices and standards. For example, the current Brazilian standard NBR 15116 [28] allows the use of recycled aggregate only in non-structural concrete.

Thus, the objective of this research is to contribute (in the Brazilian Amazon, but not only) to the production, characterization, and use of recycled CDW aggregates in the civil construction production chain, specifically for application in Portland cement matrices such as concrete and mortars for laying and coating, promoting the sustainable development and contributing positively to the formulation of management plans for a correct and efficient final disposal of the construction and demolition waste.

## 2. Materials and Methods

### 2.1. Experimental Plan

The research was carried out in the urban perimeter of Rio Branco, capital of the state of Acre, Brazil. Rio Branco is one of the largest cities in the Brazilian Amazon region, with an estimated population of 413,418 inhabitants in 2020 [29].

**Figure 1:**
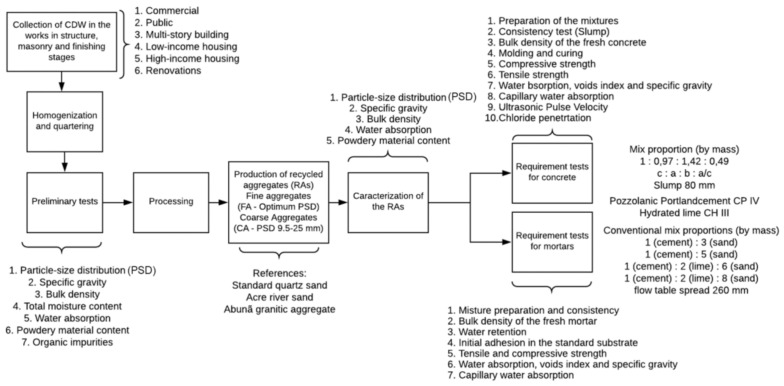
Flowchart of the adopted methodology.

The research consisted of collecting the CDW samples in the municipality’s construction sites, observing the construction typology and the execution phases. Then, the physical characterization of the collected material, processing for the production of recycled aggregate, physical characterization of the processed material and technical tests of application requirements for concrete and laying/coating mortars. Figure 1 presents a flowchart of the adopted methodology.

### 2.2. Information on the Construction Sites

Firstly, the sites (addresses) and the licensed areas for construction were obtained from the Regional Council of Engineering and Agronomy of the state of Acre (CREA-AC from the initials in Portuguese), through the Open Technical Responsibility Notes (ART’s from the initials in Portuguese), in order to identify the works in progress and make it possible to determine the collection and composition of crushed CDW (Class A) [30]. These data are not public and were provided at the request of the Federal University of Ouro Preto.

The collection and composition of the CDWs were designed based on six building types (proposed by the authors): low-income housing, high-income housing, multi-story building, commercial building and public building, and renovations. Apart from the buildings listed under “renovations”, all others were considered new constructions.

### 2.3. Sampling

CDWs are classified from A to D according to Brazilian standard NBR 15113 [31] as follows:

- Class A: mortar, concrete, and ceramic components (e.g., bricks, blocks, tiles, and coverings);
- Class B: plastics, paper, cardboard, metals, glass, and wood;
- Class C: plaster-based residues; and
- Class D: hazardous waste, such as paints, solvents, and oils.

The collection of crushed CDW (Class A) was carried out for each typology at four construction sites according to each execution stage, totaling 72 points of collection waste, 12 for each constructive typology. The execution stages included the following: (a) structures, composed of the execution of infrastructure and superstructure; (b) masonry, composed of the execution of vertical fences, mortar coatings, and counter-floors; and (c) finishing, which included installations in general, ceramic tiles, roofing, painting, and final services.

After selecting the construction sites, periodic visits began, collecting the samples taken at various points of the stationary collection bucket or the waste piles at the construction sites, being properly packed in an enclosure, and avoiding contamination, obeying the collection and storage requirements prescribed by the standard NBR 10007 [32]. The visits took place between May/2018 and April/2019. Each site was visited 24 times a week. The waste was collected with the authorization of the construction companies responsible for each construction site, kindly following the request of the Federal University of Ouro Preto.

**Figure 2:**
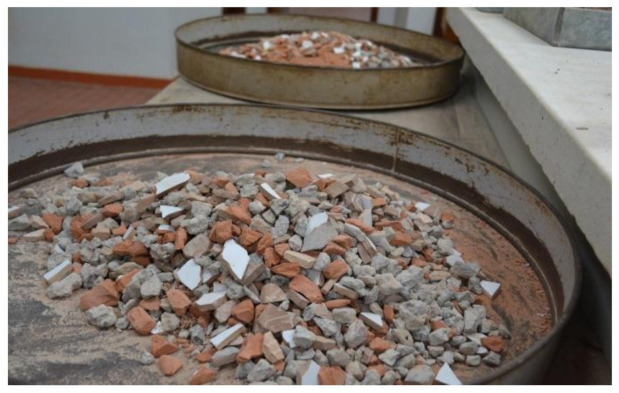
Aspects of a sample of CDW from a construction site after separation by type and particle size.

Additionally, 24 samples of CDW were collected at each site to determine its composition. With approximately 200 dm³, the samples were obtained with a shovel at random points inside the skips. They were subsequently taken to the laboratory of the Federal University of Acre for homogenization and quartering. Figure 2 shows the aspect of a sample after separation by type and particle size range.

### 2.4. Characterization of the CDWs and Production of the Recycled Aggregates

The CDW samples were submitted to an initial characterization program comprising of:

(i) Particle-size distribution (PSD) (NBR NM 248 [33]): The dry samples were placed in a set of sieves ranging from 75 mm to 75 µm, promoting the mechanical agitation of the set, for a reasonable time to allow the separation and previous classification of the different grain sizes.

(ii) Specific density (NBR NM 52 [34]) and (NBR NM 53 [35]): The fine aggregate was immersed in water until complete saturation for 24 h; then, it was disposed on a flat surface, subjecting it to the action of a gentle air current, revolving the sample frequently until the fine aggregate grains showed no strong adherence between them, which is observed by the collapse of a molded cone trunk measuring (40 ± 3) mm in diameter, (90 ± 3) mm in diameter and (75 ± 3) mm in height. The mass was determined in this condition (saturated surface dry). Then, 500 g of the material was used to assess the specific mass in a 500 cm³ pycnometer. The dried coarse aggregate with known mass was immersed in water until complete saturation for 24 h and then wrapped with an absorbent cloth until the elimination of visible water, recording the mass of the material in the saturated surface dry condition. The hydrostatic weight was measured.

(iii) Bulk density (NBR NM 45 [36]): The dry samples were placed in a calibrated mold (with known volume), in 3 layers, compacted with 25 strokes per layer by a punch rod with a semi-spherical end (16 mm × 600 mm). After that, the mass of the set was recorded.

(iv) Moisture content (NBR 9939 [37]): The masses of the samples were recorded and then oven-dried to record the dry mass.

(v) Water absorption (NBR NM 30 [38] and NBM NM 53 [35]): The saturated surface dry condition of the fine aggregates was determined as described in (ii). After that, the samples were oven-dried to determine the dry mass. The dried coarse aggregate with known mass is immersed in water until complete saturation for 24 h. Then, the saturated surface dry condition is obtained as also described in (ii).

(vi) Powdery material content by (NBR MN 46 [39]): The method consists of determining, by washing, the amount of material thinner than the opening of the 0.075 mm sieve mesh present in coarse or fine aggregates.

(vii) Organic impurities by (NBR NM 49 [40]): 200 g of the dry aggregates were placed in Erlenmeyer flasks, adding 100 mL of sodium hydroxide solution (3%). A standard solution with 3% tannic acid dissolved in a sodium hydroxide solution was also prepared in another bottle. The solutions were shaken vigorously and left for 24 h. Then, the samples were filtered, and their colors were compared.

After being characterized, the residues were manually crushed and separated by size (sieving) for the production of recycled fine and coarse aggregates, adopting the particle size distribution curves corresponding to the optimum range (for fine aggregates) and the range 9.5–25 mm (for coarse aggregates), according to the specification of the Brazilian standard NBR 7211 [13]. Then, the recycled aggregates were submitted to the initial characterization program again (except for moisture and organic impurities). The fine and coarse recycled aggregates produced were identified according to the samples’ composition and related construction stage, as shown in Table 1.

The conventional aggregates used as a reference for comparison purposes were (a) standard quartz sand (SQS) from the Technological Research Institute (IPT from the initials in Portuguese); (b) acre river sand (ARS); and (c) the granitic coarse aggregate from the Abunã region (AGA). These last two are used in construction works in Rio Branco. Adopting the IPT’s SQS is justified because it has the same particle size distribution curve of the CDW recycled fine aggregates (RFA), differently from the ARS.

### 2.5. Requirement Tests for Mortars

The requirement tests for mixed mortars for laying and coating were carried out, comprising:

(i) Preparation and measurement of the consistency index (NBR 13276 [41]): The mortars were prepared in a Fortest standard mechanical mixer (model VC 370, 3/4 HP) adopting 30 s at low speed, 60 s interval to scrape the container and 30 s at high speed. A portion of mortar is molded, filling a conical trunk mold on a flow table. Then, thirty drops in a time interval of 30 s are applied, and the spread is measured. In this work, the spreading was fixed at 260 mm ± 5 mm.

(ii) Bulk density (NBR 13278 [42]): Mortars were prepared according to the proposed treatments and placed in a known volume mold, and the mass of the set was recorded.

(iii) Water retention NBR 13277 [43]): The previously prepared mortar was introduced into a mold, and the mass of the set was recorded. Two gauzes were placed on the mortar surface, along with a set of filter papers. Then, a rigid plate was centrally placed above the set, applying a weight of 2 kg for 2 min. Then, the mass of the set of wet filter papers was measured to determine the water absorbed.

(iv) Initial adhesion to the standard substrate [44]: consisted of preparing and weighing approximately 1L of mortar, throwing the mortar against a standard substrate with a 265 cm² trowel from a distance of 30 cm in five approximately equal amounts, and weighing the rebounded material (not adhered to the substrate). The result consisted of calculating the percentage of material adhered to the substrate in relation to the material that was actually launched.

(v) Compressive strength and tensile strength in flexion (NBR 13279 [45]): For each treatment, 40 mm × 40 mm × 160 mm prismatic specimens (CPs) were tested at 28 days of age. An EMIC press, model DL 20000, equipped with a 20 KN load cell, was used. The flexural strength test consisted of applying a load ratio of 50 ± 10 N/s in the center of the bi-supported specimens with a spacing of 10 cm between the supports until the rupture. The compressive strength tests were performed using the halves of the prismatic specimens broken in the flexural tests applying a load ratio of 500 ± 50 N/s.

(vi) Water absorption, voids index, and specific gravity (NBR 9778 [46]): The specific mass, water absorption, and voids index tests were performed according to NBR 9778, equivalent to ASTM C642, with adapted samples. The test procedure includes oven-drying the samples at 105 °C for 72 h to measure dry weight; submerging the samples in water for 72 h, and boiling them for 5 h to obtain the saturated weight; and, subsequently, hydrostatic weighing of the samples.

(vii) Water absorption by capillarity (NBR 9779 [47]): Cylindrical specimens ∅5 cm × 10 cm (diameter × height) were placed in a container filled with water with their bottom constantly in contact with a 5 mm height water layer. The sample masses were determined at 3, 6, 24, and 48 h to obtain the absorption masses by capillary rise.

A Portland pozzolanic cement (specified in Brazil as CP IV-32 type) and hydrated lime (specified in Brazil as CH III type) were used to produce the mortars. Four usual mix proportions (cement:hydrated lime:sand), commonly used in Rio Branco, were adopted in the tests; they are (i) 1:0:3; (ii) 1:0:5; (iii) 1:2:6; and (iv) 1:2:8. Three types of sand were used, they are (i) the standard quartz sand (SQS); (ii) the acre river sand (ARS); and (iii) the ten sub-types of recycled sands (RS) listed in Table 1, comprising 48 mortar samples and four specimens for each test. The mortars were produced for fixed workability to evaluate the properties, with the consistency index set at 260 ± 5 mm on the flow table test. The adopted nomenclature used for the mortars consists of the acronym corresponding to the fine aggregate (Table 1) followed by the mix proportions. For example, SQS103 is the nomenclature adopted to the mortar produced with the standard quartz sand with the mix proportions 1:0:3 (cement:hydrated lime:sand).

### 2.6. Requirement Tests for Structural Concrete

The requirement tests for the C25 structural concrete (25 MPa) were carried out, comprising:

(i) Mixture preparation (NBR 12655 [48]): The concretes were prepared in a conventional concrete mixer to obtain a homogeneous mass. The mixing order was coarse aggregate; 1/3 of the water; cement; 1/3 of the water; fine aggregate; 1/3 of the water. The mixing times were 30 s for the first mixtures and 5 min for the final homogenization.

(ii) Slump test (NBR NM 67 [49]): For each sample, a conical mold (100 mm × 200 mm × 300 mm) was filled with concrete in 3 layers and then compacted with 25 uniform strokes of a punching rod with a semi-spherical end (16 mm × 600 mm). After removing the mold, the concrete slump height was determined. In this research, the targeted value was 80 mm ± 10 mm.

(iii) Bulk density in the fresh state (NBR 9833 [50]): The concretes were placed in a known volume mold, in 3 layers, compacted with 25 strokes per layer by a punching rod with a semi-spherical end (16 mm × 600 mm), and the mass of the set was recorded.

(iv) Molding and curing (NBR 5738 [51]): The concretes were cast in cylindrical molds measuring ∅10 × 20, being compacted in two layers, with 12 strokes per layer, by a punching rod with a semi-spherical end (16 mm × 600 mm). After 24 h, they were demolded and kept in a humid chamber for 28 days at a temperature of 23 ± 2 ° C and relative humidity of 95%.

(v) Compressive strength (NBR 5739 [52]) and (vi) tensile strength (NBR 7222 [53]): Cylindrical specimens measuring ∅10 cm × 20 cm (diameter × height) were tested at 28 days of age. For the mechanical tests (compressive strength and tensile strength by diametrical compression), the hydraulic press EMIC DL 20000 was used, with a load cell of 200 kN (compression) and 20 kN (tension) with a loading speed of 0.3 MPa/s (compression) and 0.05 MPa/s (tension).

(vii) Water absorption, voids index, and specific density (NBR 9778 [46]): The specific mass, water absorption, and voids index tests were performed according to NBR 9778, equivalent to ASTM C642, with adapted samples. The test procedure includes drying the samples in the oven at 105 ° C for 72 h to measure dry weight; submerging the samples in water for 72 h and boiling them for 5 h to obtain the saturated weight; and, subsequently, hydrostatic weighing of the samples.

(viii) Capillary water absorption (NBR 9779 [47]): Cylindrical specimens measuring ∅10 cm × 20 cm (diameter × height) were placed in a container filled with water with its base constantly in contact with a 5 mm height water layer. The sample masses were determined at 3, 6, 24, and 48 h to obtain the absorption masses by capillary rise.

(ix) Ultrasonic pulse velocity (UPV) (NBR 8802 [54]): A Proceq TICO device was used set to 54 kHz p-waves. Direct measurements were made on concrete specimens measuring ∅10 cm × 20 cm (diameter × height).

(x) Accelerated chloride penetration [55]: The method based on NT Build 443 was applied. The specimens were sealed and immersed in a 2.8 M NaCl solution with one uncovered face exposed for a minimum period of 35 days.

The Brazilian Portland pozzolanic cement CP IV-32 (Itaú Votorantim) was also used to produce the concretes. The mix design method of the Brazilian Portland Cement Association [56] was used, leading to the proportions (by mass) of 1:0.97:1.42:0.49 (cement:sand:gravel:water/cement). Partial (50%) and total (100%) replacement of conventional aggregates by recycled aggregates were proposed and evaluated (Table 1). Due to the high absorption rate of recycled aggregates, research by Rodrigues and Fucale [57], Seara-Paz et al. [58], González-Fonteboa et al. [59], Lovato et al. [60], Agrela et al. [61], and Zaharieva et al. [62] emphasized problems in the workability and the alteration of the water/cement ratio of the concrete, causing a reduction in compressive strength. To address this drawback, it is necessary to pre-wet the recycled aggregates to reach adequate workability, avoiding strength losses. Thus, in this research, the recycled aggregates were pre-watered at 50% of the absorption capacity. Finally, the concretes were designed for a slump fixed at 80 mm ± 10 mm and a plasticizer admixture based on sulfonated salts and carbohydrates (Sika Concreto Forte) was used to adjust the workability. Twenty-one concrete samples were produced, and four specimens were molded for each test.

The adopted nomenclature used to identify the concretes consists of the acronym corresponding to the aggregate (Table 1) followed by the replacement rate. For example, S050 is the nomenclature adopted to the concrete produced with partial replacement (50%) of the conventional aggregates by recycled aggregates obtained from the structure building stage. The reference concrete (REF) was produced using the natural quartz sand from the Acre River and de Abunã granitic coarse aggregate.

## 3. Results and Discussion

### 3.1. Composition and Characterization of Collected Waste

Sorting and the correct separation of waste is fundamental to ensure the quality of the recycled aggregate. Several countries such as Denmark, The Netherlands, and Germany achieve good recycling rates due to strict environmental policies. In contrast, in other countries, the low rates are related to the lack of consumer confidence due to non-screening, generating inconsistent quality aggregates [63,64].

**Figure 3:**
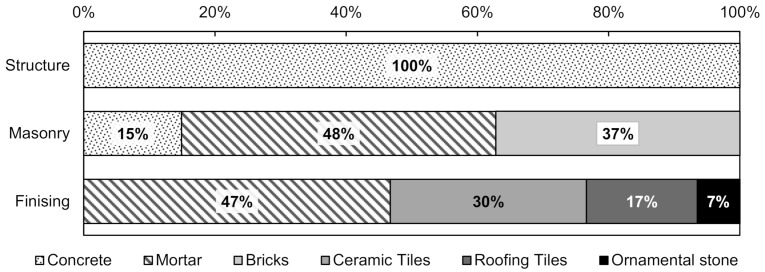
Composition of the waste collected in the construction sites.

Regarding the quality of recycled aggregates, the residues were separated by removing contaminants. The compositions of class A crushed waste collected in the construction sites consist primarily of (i) concrete in the structure building stage; (ii) mortar, bricks, and concrete in the masonry building stage; and (iii) mortar, ceramic tiles, and ornamental stones in the finishing stage. The proportions are shown in Figure 3.

The use of homogeneous recycled aggregate (similar to those obtained in the structure construction stage with 100% concrete) leads to better quality cementitious matrices. In contrast, mixed recycled aggregates generally present greater porosity, varied compositions, lower strengths, and cementitious matrices with more porous microstructure and inferior properties [65,66,67,68].

**Figure 4:**
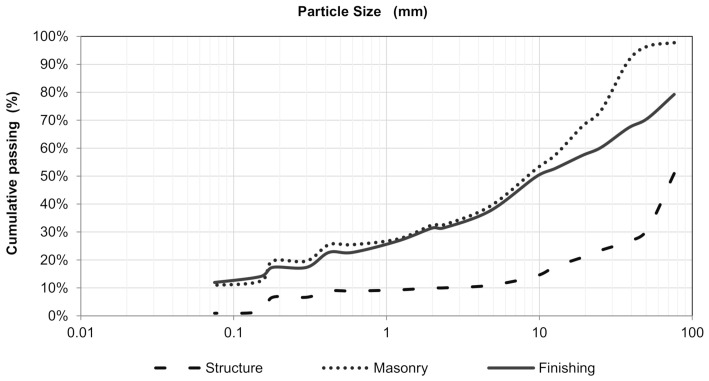
Particle size distribution curves of the waste collected in the construction sites.

**Figure 5:**
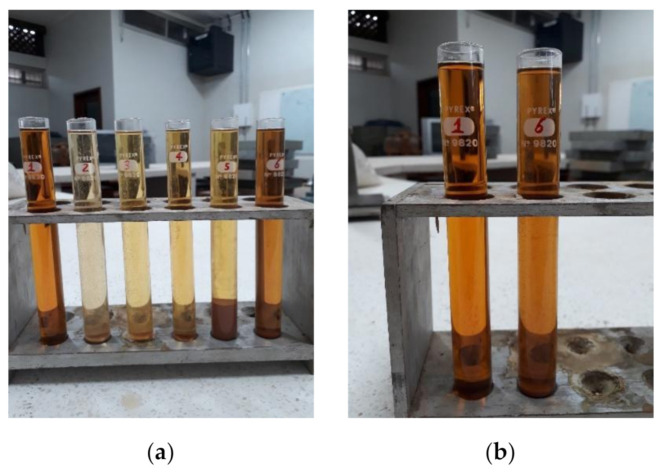
Evaluation of organic impurity of the waste collected in the construction sites: (**a**) display showing all the samples examined (reference solution of tannic acid on the left); (**b**) display showing the reference solution of tannic acid (**left**) and the solution obtained from the finishing stage residue (**right**).

Figure 4 shows the particle size distribution curves of the collected waste; Table 2 lists the characterization program results; and Figure 5 shows the results of the evaluation of organic impurities.

Except for the samples obtained from the finishing stage, the raw-waste particles are poorly graded, as observed in Figure 4 and quantified by the Cu and Cc coefficients (Table 2). The structure stage residues had the highest density and water absorption due to having a homogeneous cementitious matrix with coarse aggregate of granitic origin with a higher density than ceramics and high absorption due to being produced with sand from the river Acre, with uniform curve and grains, which contributes to a more porous matrix.

The finishing stage residues presented the lowest density, water absorption, moisture content, and bulk density due to the composition comprising 53% low-absorption materials (roofing tiles, ceramic tiles, and ornamental stone) and predominance of tabular particles (worst grain packing). In contrast, masonry residues had the highest bulk density and moisture content due to the porosity of bricks and laying mortars and for presenting more equant particles that improve the grain packing.

The samples of SQS, ARS, residues from the structures stage, and the masonry stage showed lighter colors than the standard reference sample (solution of tannic acid). In contrast, the sample obtained from the finishing stage presented a color equal to the reference (highlighted in Figure 5b) due to organic admixtures in adhesive mortars. Based on the results, all the samples met the maximum acceptable limits for harmful substances in the aggregates, according to NBR 7211 [13].

### 3.2. Composition and Characterization of Collected Waste

**Figure 6:**
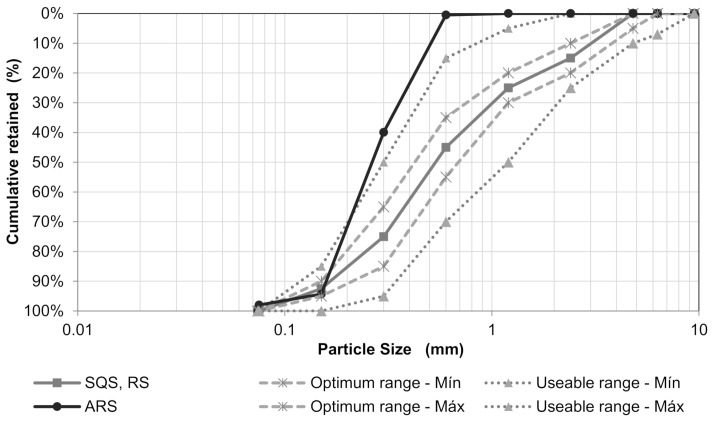
Small aggregate size range.

To carry out the tests of technical requirements for application in mortar and concrete, the RFAs were produced, and the SQSs were blended to meet the optimum particle-size range of the Brazilian standard NBR 7211 [13] (Figure 6).

The RFA and the SQS have a maximum characteristic diameter of 4.75 mm and a fineness module of 2.53, where we observe a well-graded curve. The particle-size curve of the ARS is almost entirely outside the lower usable range limit, classifying it as very thin, with a maximum characteristic diameter of 0.6 mm and a fineness module of 1.35. The characterization of recycled post-processing aggregates is shown in Table 3.

The aggregates produced in the optimum zone curve had continuous particle-size distribution, with grains that allow greater packing density into the cementitious matrix, providing mortars and concretes with better quality than aggregates that present uniform granulometry, such as the ARS [69,70,71].

In general, the recycled aggregates presented lower densities, lower bulk densities, higher powdery material content for the fine aggregates, and greater water absorption than the conventional aggregates (SQS, ARS, and AGA). In general, these are common characteristics, as they have been reported by several authors [19,72,73,74].

The densities of the residues are very close. The masonry stage recycled aggregates had the highest density, which was followed by the structure stage recycled aggregates. The finishing stage recycled aggregates had the lowest density. The structure stage residues presented the highest bulk density, water absorption, and powdery material content for recycled fine aggregates. In contrast, masonry presented the lowest water absorption and content of powdery material. The finishing waste had the lowest bulk density. In general, as porosity increases, its ability to absorb water increases and its density decreases [75].

For recycled coarse aggregates, masonry had the lowest bulk density and the highest water absorption. In contrast, the finishing waste had the highest bulk density and the lowest water absorption due to the composition of impermeable materials that allow less absorption. In general, recycled aggregates’ outstanding properties make it possible to obtain cementitious matrices of lower densities, higher voids, and water absorption [76,77,78,79,80].

**Figure 7:**
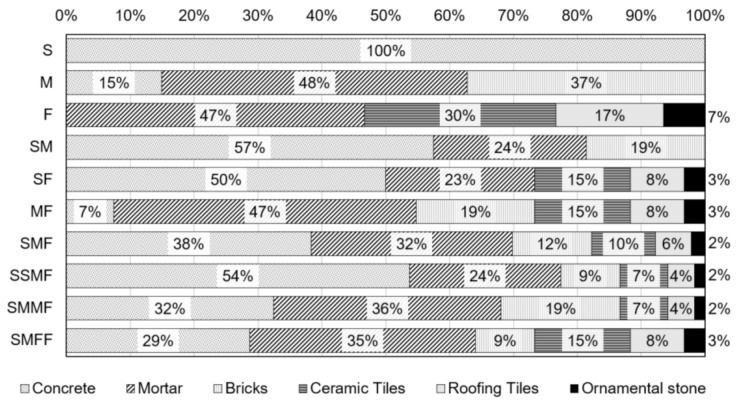
Gravimetric composition of the processed samples.

The gravimetric composition of the samples used in the requirements tests for application in laying/coating mortars and concretes are presented in Figure 7.

The waste compositions make it possible to detach the samples with the best physical and mechanical properties suitable for use in civil construction. The recycled aggregates (structures, masonry, and finishing) are composed of concrete (structures), mortar, brick and concrete (masonry), and mortar, ceramic tile, and ornamental stones (finishing). For the blends, concrete and/or mortar predominated followed by ceramic residues.

### 3.3. Requirement Testing for Laying/Coating Mortars

**Figure A1:**
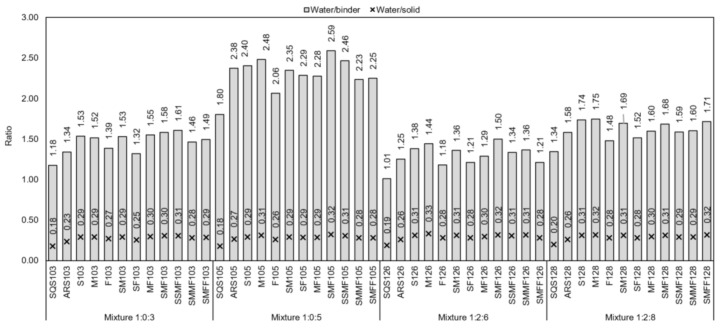
Water/solid ratio and water/binder ratio.

The mix preparation tests showed that the mortar samples containing recycled fine aggregate had a higher water/solid factor (water mass/anhydrous mass) and a higher water/binder ratio than the reference mortars (produced with conventional aggregates) (Appendix A Figure A1). This effect occurs because recycled fine aggregates are more porous, requiring more water to achieve the same workability due to the greater absorption [19,72,73,74].

**Figure A2:**
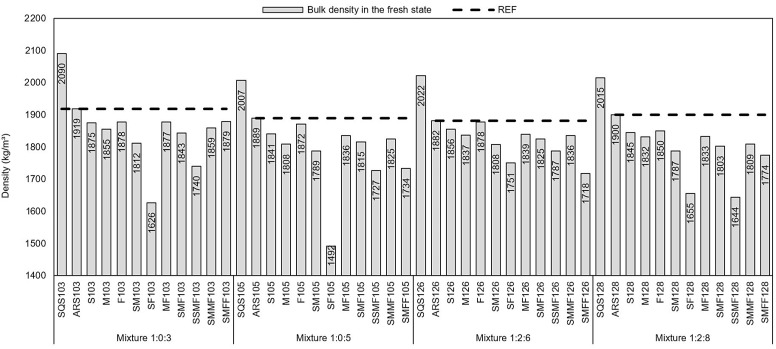
Bulk density in the fresh state.

**Figure A3:**
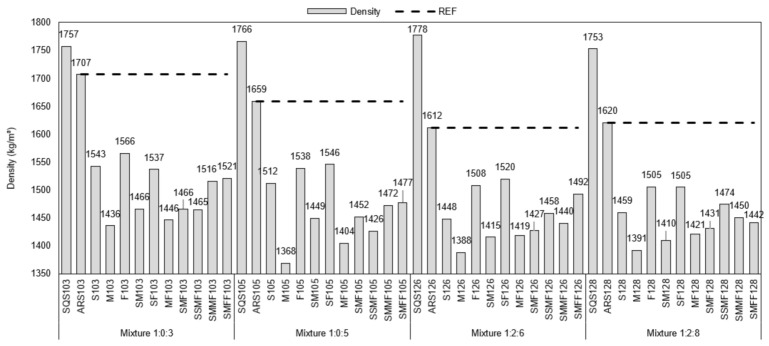
Density in the hardened state.

Bulk densities in the fresh state were between 1492 and 2091 kg/m³ so that the mortars were classified as normal density mortars (1400 ≤ ρ ≤ 2300) [81] (Appendix A Figure A2). In the hardened state, the densities varied between 1368 and 1778 kg/m³ (Appendix A Figure A3).

Mortars with recycled aggregates, both fresh and hardened, showed lower densities than mortars with conventional aggregates due to the increase in the voids index and the use of aggregates with lower densities, corroborating with the same relationship for the densities of the aggregates [82].

**Figure A4:**
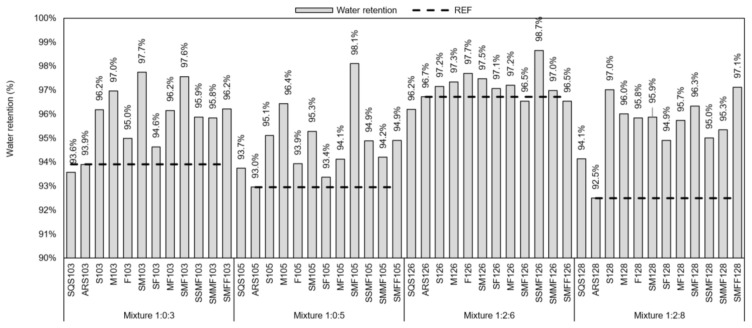
Water retention.

The mortars had water retention higher than 92% (Appendix A Figure A4), which is higher than the requirement of ASTM C-270 [83] of at least 75%. Mortars with recycled fine aggregates had better results compared to mortars with conventional aggregates, meaning that waste mortars had a greater capacity to maintain their workability in the fresh state when subjected to water loss and still allowing better mechanical strength, adhesion, and durability in the hardened state due to greater interference in the chemical reactions of the binders that require an adequate amount of water [81].

**Figure 8:**
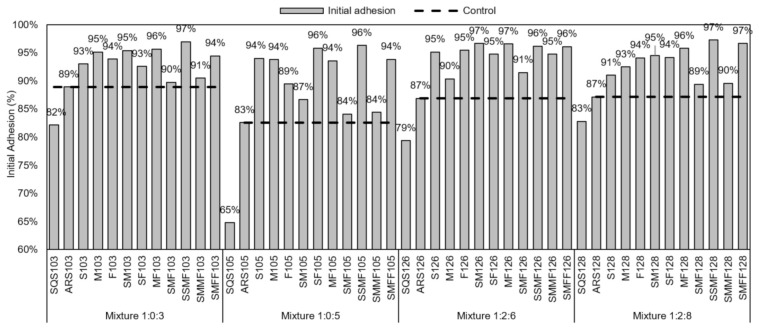
Initial adhesion of mortars to the substrate.

The results of the initial adhesion tests of fresh mortars are shown in Figure 8.

The results varied between 65% and 97%, emphasizing that the mortar samples produced with recycled fine aggregates had higher initial adhesions than the mortars with conventional aggregates, allowing less waste and generation of residues.

**Figure 9:**
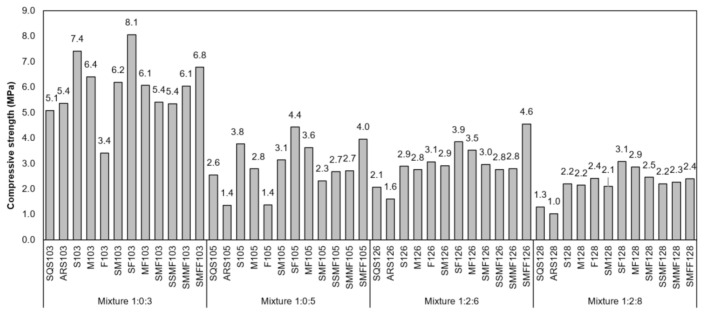
Compressive strength of the mortars.

**Figure 10:**
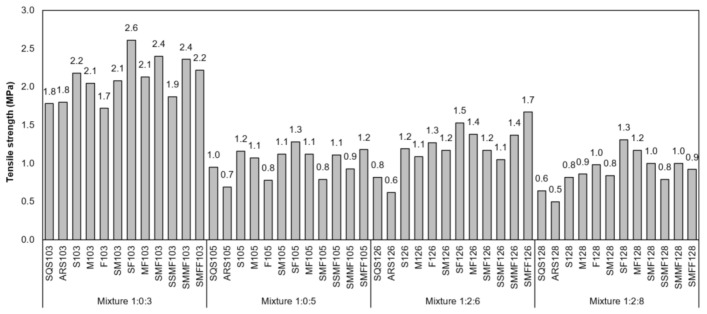
Tensile strength of the mortars.

In this research, the authors proposed the production and evaluation of mortars for laying and coating, using mix proportions consolidated in the local construction industry. These mortars present modest mechanical performances compared to structural concrete since the main technological properties of interest are workability, water retention, and initial adhesion in the fresh state, small dry shrinkage, and adhesion strength, lack of cracks, permeability, and ductility in the hardened state. The CDW mortars showed better mechanical results in the compression (Figure 9) and tension (Figure 10) strength tests than the conventional mortars, except for the F103 mortar, which was produced with aggregates produced from the finishing residues. The mortars were classified in the range P1 to P6 in compressive strength and R1 to R3 in tensile strength (NBR 13281 [84]).

The gains in compressive strength reach values of up to 225% (SF105), which are considerably higher than mortars currently used in construction works in Rio Branco. The tensile strengths in flexion were slightly higher and closer to the average strengths, showing that the CDW mortars had better performance under traction and a higher adherence, giving them a better behavior to temperature changes and hygrothermal variations.

The increases in strengths observed in mortars with CDW recycled aggregates are related to the presence of non-hydrated cement in the grains of cementitious residues, greater compactness with the packing of the grains provided by a well-graded particle size distribution, and the greater water retention interfering in the chemical reactions of the binders [22,85,86]. Mortars containing recycled aggregates showed better mechanical performances than conventional mortars. The fine aggregates with continuous particle size distributions and a greater amount of fines reduce segregation and help to achieve denser grain packing and a less porous matrix [75,87,88]. In this sense, as the recycled aggregate has greater water absorption, there may be greater adherence between the paste and the aggregate by absorption of the paste and precipitation of the hydrated crystals into the aggregate’s pores [89,90]. Thus, we found that CDW mortars make it possible to reduce binder consumption, resulting in savings and better sustainability performance than conventional aggregates.

**Figure A5:**
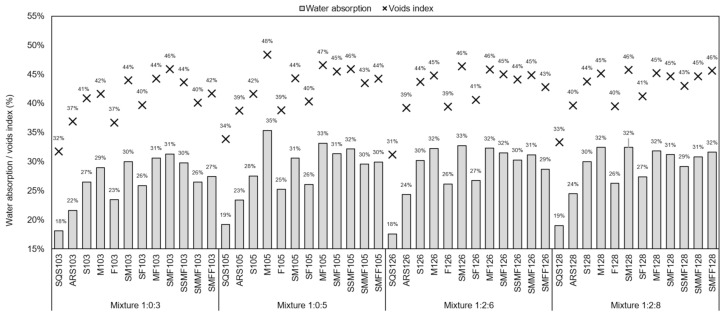
Water absorption.

**Figure A6:**
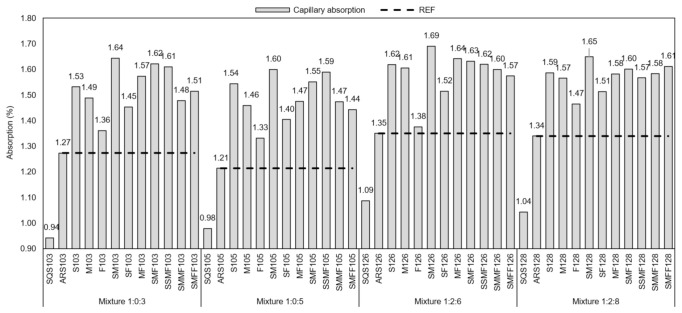
Capillary absorption.

The CDW mortars showed superior results of voids content, water absorption by immersion, and capillarity in the hardened state compared to the conventional mortar with ARS, being up to 9.6% higher for the voids index (M105), 9.7% higher for water absorption by immersion (SMF103), and 32.23% higher for capillary water absorption (SM105) (Appendix A Figure A5 and Figure A6).

In general, the replacement of natural aggregates by CDW recycled aggregates in mortars requires more water to maintain the same workability. Despite this, the recycled mortars had greater strength to compression and traction, greater adhesion to the substrate, lower density, and greater water absorption by immersion and capillarity [76,77,78,79,80,91]

### 3.4. Requirement Testing for Concrete

**Figure A7:**
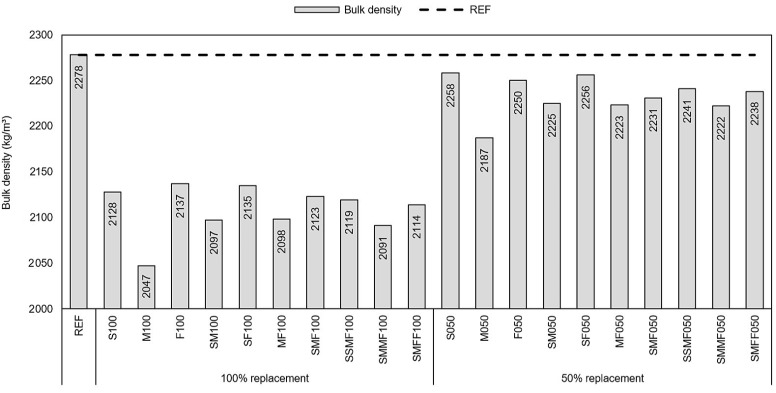
Bulk density in the fresh state.

**Figure A8:**
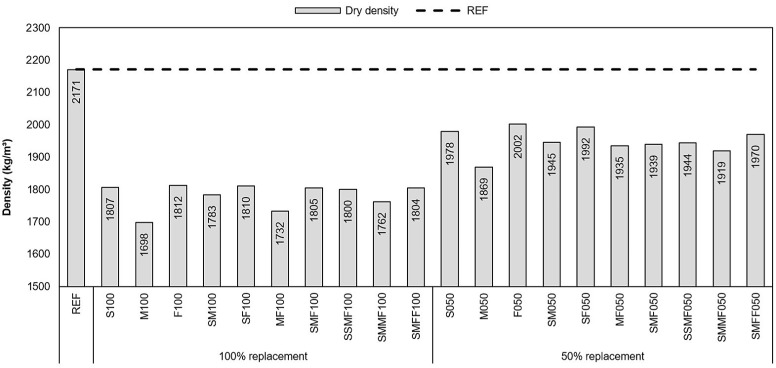
Dry density.

All the concretes had bulk densities in the fresh state between 2047 and 2278 kg/m³ (Appendix A Figure A7). In the hardened state, the dry density ranged from 1698 to 2171 kg/m³ (Appendix A Figure A8). Concretes with recycled aggregates, both fresh and hardened, showed lower densities than concretes with conventional aggregates; this is due to the incorporation of air and the consequent increase in voids, corroborating the same relationship for the densities of the aggregates [57,92,93,94].

According to NBR 8953 [95], we can classify the reference concretes and the F050 as normal concretes with dry bulk density between 2000 and 2800 kg/m³. All the other concretes produced with the CDW recycled aggregates were classified as lightweight concretes with dry densities lower than 2000 kg/m³. That is a positive characteristic since reducing the parts’ weight positively influences the structural dimensioning.

**Figure 11:**
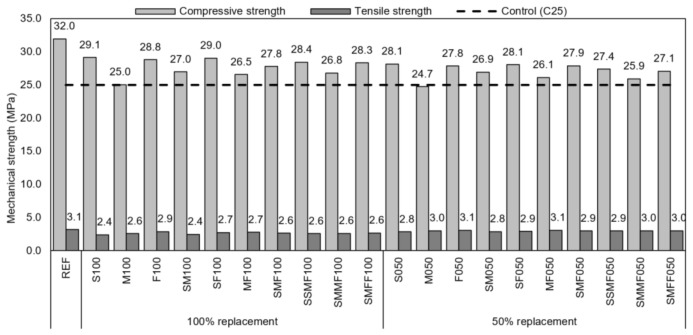
Mechanical strength of the concretes.

The reference concrete presented a superior mechanical performance compared to the recycled concrete, which corresponded to 86% of the compressive strength and 88% of the tensile strength of the REF, on average (Figure 11). Chen, Yen, and Chen [24] obtained values around 90% of the reference concretes’ compressive and flexural strength. According to Vázquez et al. [96], the loss in compressive strength is up to 20% for 100% replacement and 2% to 15% for 50% replacement. Etxeberria et al. [97] reported losses of 20 to 25% in compressive strength for 100% replacement, maintaining the w/c ratio and cement content.

From the point of view of performance, the results were positive, since all the concretes were designed to meet the compressive strength of 25 MPa. All but the M050 (24.7 MPa) achieved a compressive strength equal or superior to the goal.

Conventional concretes produced with conventional aggregate showed greater strength compared to concretes produced with CDW aggregates. On the one hand, the improved mortar fraction is beneficial to the strength of the concrete by improving the interface transition zone and increasing the stiffness of the matrix [75,87,88]. On the other hand, recycled coarse aggregates are weaker than rocky aggregates, limiting the concrete’s strength. Additionally, CDW concretes have higher w/c ratios due to the high porosity of the recycled aggregates, which increases the matrix porosity [89,98].

The decrease in concretes’ strength with recycled aggregates is mainly due to the change in the w/c ratio [99]. Packing effects occur with the fine aggregate, increasing the stiffness of the matrix; however, with the coarse aggregate, although there is also an improvement in the transition zone, the grain of the aggregate is more fragile in comparison to the matrix, and the failure can occur in the aggregate due to its larger dimension [89].

The concrete with aggregates obtained from the structures building stage presented the best result among the recycled concretes due to the aggregates’ nature being made of concrete; therefore, it was homogeneous and of better quality than the other phases’ heterogeneous aggregates constituted by mixed ceramic aggregates and mortar.

The strengths of recycled concretes can be significantly improved by non-hydrated cement present in the grains of residues obtained from cementitious composites, as observed in the coating mortars.

**Figure 12:**
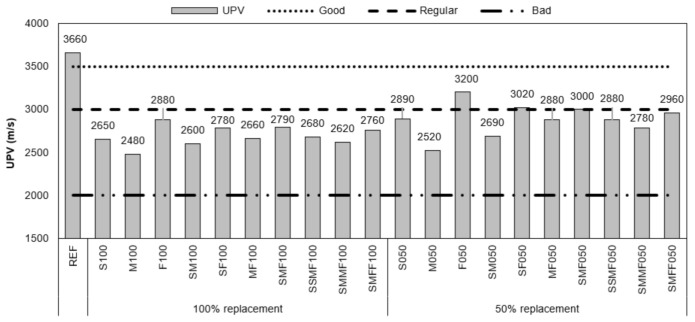
Results of the ultrasonic pulse velocity measurements in the concretes.

The results of ultrasonic pulse velocity (UPV) in concretes showed that the reference concrete’s quality was evaluated as good (Figure 12), while the CDW recycled aggregate concretes F050, SF050, and SMF050 were classified as regular according to the British standard EN 12504-4 [100]. All the other recycled concretes were classified as bad with proximity to regular.

The ultrasonic pulse velocity is used to evaluate the concrete’s uniformity, and as the ultrasonic wave propagates more quickly in dense media than in the air, the more pores there are and the smaller the sample stiffness, the longer the ultrasonic pulse propagation time. This fact explains the reduction in UPV in recycled aggregate concretes, as they present lower density, greater porosity and greater water absorption compared to reference concrete, leading to a longer travel time for the ultrasonic wave [101,102,103,104].

Considering that the VPU corroborates with the data obtained from the mechanical tests, we emphasize that even for concretes qualified as regular or bad by the British standard using the uniformity criterion, the recycled concretes still present satisfactory results of mechanical strength for use in civil construction.

**Figure A9:**
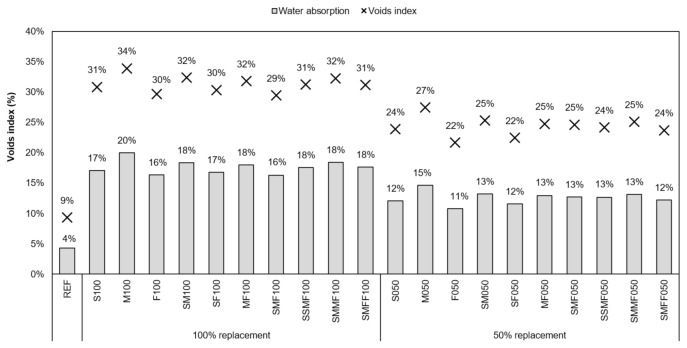
Water absorption and voids index.

**Figure A10:**
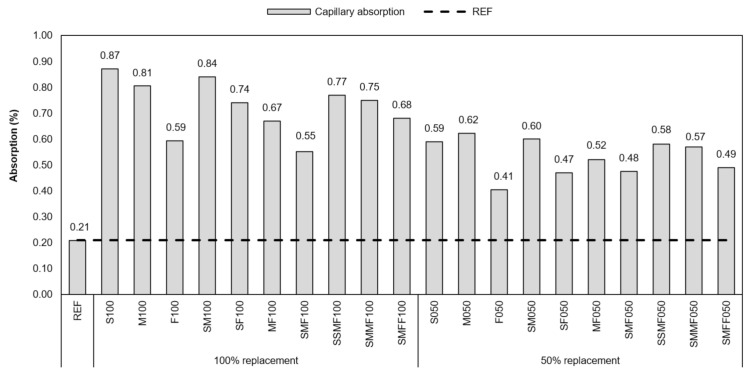
Capillary absorption.

The results of the measurements of voids content, water absorption by immersion, and capillarity in the hardened state showed that the waste concrete presented higher values compared to the REF conventional concrete, being up to 33.93% higher for the voids index (M100), 19.98% for water absorption by immersion (M100), and four times for capillary water absorption (S100) (Appendix A Figure A9 and Figure A10). The concretes with total replacement of conventional aggregates by CDW recycled aggregates showed higher values than those of partial substitution, as expected, and these were higher than the reference concrete.

**Figure 13:**
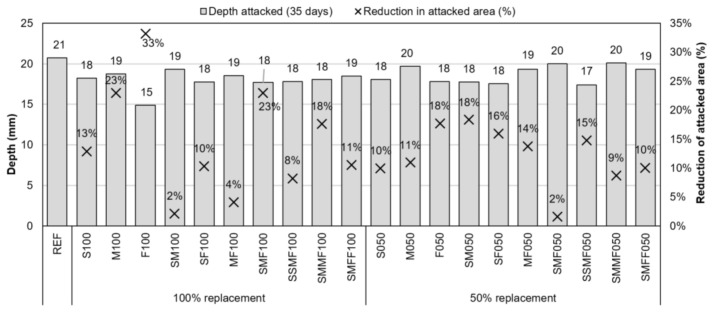
Depth and reduction of the area attacked by chlorides after 35 days of exposition to a 2.8 M NaCl solution.

The specimens were sealed for the chloride attack test, and one uncovered face was exposed to a 2.8 M NaCl solution for 35 days, achieving penetration depths up to about 2 cm. All the concretes with CDW recycled aggregates presented values inferior to the reference concrete regarding depth and area attacked by chloride (Figure 13). The F100 concrete presented the lowest value of depth attacked by chloride of 14.9 mm and the highest value of reduction of attacked area of 33.2% compared to the reference area. The concrete SMMF050 presented the highest depth value of 20.1 mm close to and below the reference concrete (20.8 mm). The concretes with the lowest reduction in the attacked area were SMF050 (1.6%) and SM100 (2.1%).

The concrete produced with recycled CDW aggregates showed greater durability than conventional concrete due to the continuous particle size distribution of the recycled fine aggregate and a greater amount of fines that contribute to improving the packing density, reducing voids between the grains and making it difficult for chloride ions to migrate into the concrete. On the contrary, the ARS presents uniform particle size distribution and expected worst packing performance.

Similar depths of water-soluble chloride penetration can be expected in conventional and recycled concrete types with the same w/c ratio and curing treatment when w/c is greater than 0.40 [105]. Olorunsogo and Padayachee [106] concluded that the concretes obtained from CDW recycled aggregates had improved durability properties, decreasing chloride ions’ conductivity at certain levels of substitution and with increasing curing time.

### 3.5. Recycling Proposal

The CDW generated in the construction sites is taken to the inert landfill in Rio Branco, which has a deposition area of 13 hectares (130,000 m²). In operation since 1996, the CDWs are landfilled with no reuse or recycling process and accumulates tailings at the height of 18 m due to the operating model [107].

CDW generation rates of 450 t/day (in 2015) imply a landfill cost of US$ 300,000/year [107]. The authors estimate that more than 3 million tons of waste were disposed of at the landfill, generating a cost of US$7.5 million in 25 years of operation that could be better used if recycled. For example, the amounts spent corresponds to the construction of 555 social housing units, or the construction of 6 public schools, or the paving of 63 km of production lines in the primary sector in Rio Branco [108,109,110].

Considering crushable CDW generation rates of 355 t/day and 0.37 t/year∙inhabitant (in 2015) [107], it is currently estimated an index of 419 t/day of shredded CDW, considering a constant generation rate per inhabitant. The authors propose the design of an industrial unit for the production of construction materials from CDW, which must consider meeting the available demands. Thus, a recycling plant is proposed operating at 60% efficiency, a 22-day/month workload, and 8 h of daily production, using equipment with a processing capacity of 120 t/h with a production of gravel, sand, and pebbles to be sold for US$ 6/ton, up to 8 times cheaper than the prices of natural aggregates [12].

In a city located in the Amazon region with a lack of natural and quality aggregates, the impact of this work is to present viable alternatives with application in cementitious matrices such as Portland cement concrete and mixed mortars for laying and coating, minimizing environmental, economic, social, and technological drawbacks for the region impacted by non-adequate waste disposal.

This work encourages public policies to implement a CDW recycling plant producing recycled aggregates, enabling the reinsertion in the civil construction production chain. Allowing as a strategy to invest in social technologies that encourage social and environmental development, mitigating the generation of construction waste with incentive policies with the training of the civil construction workforce, the application of recycled aggregates in local productive arrangements, construction works/renovation and donation to low-income families for the construction of social housing and for paving production lines in the primary sector.

## 4. Conclusions

The present work characterized the application of construction and demolition waste (CDW) in the city of Rio Branco, in the Brazilian Amazon region, specifically in Portland cement matrices such as concrete and mixed mortar for laying and coating. The main findings are summarized below:

- Recycled aggregates from construction and demolition waste had lower densities and greater water absorption compared to conventional aggregates such as sand from the river Acre and granitic gravel from the Abunã region;
- The mortars produced with recycled aggregates showed better performances when compared to conventional mortars and had greater water retention; initial adhesion; compressive strength; tensile strength; absorption by immersion and capillarity; and voids index when compared to conventional mortars;
- Concretes produced with recycled aggregates showed lower densities both in the fresh and hardened state; greater water absorption; higher voids index; lower mechanical strength, both to compression and tension; lower ultrasonic pulse velocity; and greater durability to attack by chloride when compared to reference concrete with sand from the Rio Acre and gravel from the Abunã region;
- The concretes produced with recycled aggregates showed properties that allow reinsertion in the civil construction works in Rio Branco compared to the concretes produced with conventional aggregates.

The methodology with an observation of residues’ composition from the execution stages was valid and effective for this research. The mixture of residues between phases allowed the best performance of mortars and concretes to be observed. Thus, public policies may be directed to the exact application of the proportion according to the total generation of waste in different construction types.

One of the main limitations of the present work was the lack of a Brazilian standard or an accepted methodology for using recycled aggregate in structural concretes and mortars. The varied compositions of waste in different cities and even seasonally in the same city show peculiarities in the research. The literature review showed that different authors adopt different methodologies, making it difficult to compare cities and even different research works in the same city.

## Figures and Tables

**Table 1:** Identification composition of the produced recycled aggregates.

Sample ID *	Composition (Construction Stage)		
	Structures	Masonry	Finishing
S	100%	-	-
M	-	100%	-
F	-	-	100%
SF	50%	-	50%
SM	50%	50%	-
MF	-	50%	50%
SMF	33.3%	33.3%	33.3%
SSMF	50%	25%	25%
SMMF	25%	50%	25%
SMFF	25%	25%	50%

* S: residue obtained from the structure building stage; M: residue obtained from the masonry building stage; F: residue obtained from the finishing stage.

**Table 2:** Results of the characterization program of the waste collected in the construction sites.

Parameter ^1^	Structure	Masonry	Finishing
CMD (mm)	76.2	50.8	76.2
FM	8.40	5.34	5.96
D50 (mm)	76.2	9.50	9.50
D10 (mm)	4.75	0.075	0.075
Cu	32.3	169.3	338.7
Cc	14.35	4.20	2.10
γ (g/cm³)	2.60	2.51	2.44
ρ (g/cm³)	1.12	1.17	0.94
w (%)	10.13	10.31	7.98
ab (%)	13.39	12.02	10.72

^1^ CMD: characteristic maximum diameter; FM: fineness modulus; D50: diameter with 50% passing; D10: diameter with 10% passing; Cu: uniformity coefficient; Cc: curvature coefficient; γ: density; ρ: bulk density; w: water content; ab: water absorption.

**Table 3:** Characterization of the recycled aggregates.

Parameter ^1^	CMD (mm)	FM	γ (g/cm³)	ρ (g/cm³)	ab (%)	PM (%)
ARS	0.6	1.35	2.51	1.41	1.46	1.77
SQS	4.75	2.53	2.48	1.64	0.54	0.43
S-F	4.75	2.53	2.39	1.27	12.51	7.30
M-F	4.75	2.53	2.40	1.25	9.99	5.31
F-F	4.75	2.53	2.30	1.22	12.43	5.98
AGA	19	0.92	2.70	1.46	0.45	-
S-C	19	0.92	2.61	0.98	16.86	-
M-C	19	0.92	2.63	0.93	17.58	-
F-C	19	0.92	2.53	1.10	8.25	-

^1^ ARS: acre river sand; SQS: IPT’s standard quartz sand; S-F: recycled fine aggregate from the structure stage; M-F: recycled fine aggregate from the masonry stage; F-F: recycled fine aggregate from the finishing stage; AGA: Abunã granitic aggregate; S-C: recycled coarse aggregate from the structure stage; M-C: recycled coarse aggregate from the masonry stage; F-C: recycled coarse aggregate from the finishing stage; CMD: characteristic maximum diameter; FM: fineness modulus; γ.: density; ρ.: bulk density; ab: water absorption; PM.: powdery material content.

## Data Availability

The data presented in this study are available in article.
